# Chronic low back pain is associated with reduced vertebral bone mineral measures in community-dwelling adults

**DOI:** 10.1186/1471-2474-13-49

**Published:** 2012-03-30

**Authors:** Andrew M Briggs, Leon M Straker, Angus F Burnett, John D Wark

**Affiliations:** 1 School of Physiotherapy, Curtin University, Perth, Australia; 2 Curtin Health Innovation Research Institute (CHIRI), Curtin University, GPO Box U1978, 6845 Perth, Western Australia; 3 University of Melbourne Department of Medicine, Royal Melbourne Hospital, Melbourne, Australia; 4 Department of Sports Science and Physical Education, Chinese University of Hong Kong, Shatin, New Territories Hong Kong; 5 School of Exercise, Biomedical and Health Sciences, Edith Cowan University, Perth, Australia; 6 Bone and Mineral Service, Royal Melbourne Hospital, Melbourne, Australia

## Abstract

**Background:**

Chronic low back pain (CLBP) experienced in middle-age may have important implications for vertebral bone health, although this issue has not been investigated as a primary aim previously. This study investigated the associations between CLBP and dual energy X-ray absorptiometry (DXA)-derived vertebral bone mineral measures acquired from postero-anterior and lateral-projections, among community-dwelling, middle-aged adults.

**Methods:**

Twenty-nine adults with CLBP (11 male, 18 female) and 42 adults with no history of LBP in the preceding year (17 male, 25 female) were evaluated. Self-reported demographic and clinical data were collected via questionnaires. Areal bone mineral density (aBMD) was measured in the lumbar spine by DXA. Apparent volumetric (ap.v) BMD in the lumbar spine was also calculated. Multiple linear regression models were used to examine associations between study group (CLBP and control) and vertebral DXA variables by gender, adjusting for height, mass and age.

**Results:**

There was no difference between groups by gender in anthropometrics or clinical characteristics. In the CLBP group, the mean (SD) duration of CLBP was 13.3 (10.4) years in males and 11.6 (9.9) years in females, with Oswestry Disability Index scores of 16.2 (8.7)% and 15.4 (9.1)%, respectively. Males with CLBP had significantly lower adjusted lateral-projection aBMD and lateral-projection ap.vBMD than controls at L3 with mean differences (standard error) of 0.09 (0.04) g/cm^2 ^(*p *= 0.03) and 0.02 (0.01) g/cm^3 ^(*p *= 0.04). These multivariate models accounted for 55% and 53% of the variance in lateral-projection L3 aBMD and lateral-projection L3 ap.vBMD.

**Conclusions:**

CLBP in males is associated with some lumbar vertebral BMD measures, raising important questions about the mechanism and potential clinical impact of this association.

## Background

Maximising peak bone mass towards late adolescence and minimising bone loss after this period are important factors in the maintenance of optimal skeletal integrity. Although a large volume of research has been dedicated to minimising bone loss towards later adulthood, particularly in the context of age-related and post-menopausal osteoporosis, maintenance of bone strength at earlier life stages is equally, if not more important, from a lifecourse perspective. Therefore, a better understanding of the potentially-modifiable events across the lifecourse which may be associated with sub-maximal bone accretion or influence bone loss is important, particularly when considering the immense population burden of bone fragility.

Low back pain (LBP) is one of the most common and significant musculoskeletal disorders experienced across the lifecourse and is most common during middle-age [1]. It represents an enormous public health issue worldwide owing to the soaring healthcare costs associated with the condition and the societal and personal burdens imposed [2]. The lifetime prevalence of LBP is around 80% for adults [3] and adolescents [4] and although in the majority of cases the experience of LBP is benign and most people regain functional capacity, pain and disability can persist [5]. For a proportion of individuals (approximately 10-20%) the experience of LBP persists beyond three months to become chronic, experienced as either a continuous or episodic course of pain [6]. In many such cases, chronic LBP (CLBP) is associated with a myriad of biopsychosocial factors which may also have implications for bone health [7].

A relationship between impaired bone health and back pain is well established in conditions such as osteoporosis, osteoarthritis and inflammatory arthritis. In these circumstances, the association between back pain and bone health may be mediated by vertebral fracture [8], hyperkyphosis [9], inflammation and joint degeneration [10] and intervertebral disc degeneration [11]. However, skeletal integrity is rarely considered in the context of non-specific CLBP experienced during middle-age, despite some evidence for an association between back pain and impaired bone health outside the context of age-related osteoporosis and osteoporotic vertebral fractures [7]. Further, although co-morbid conditions are common among individuals with LBP, impaired bone health as a co-morbidity associated with LBP has not been examined outside older age groups. Our recent review summarised the likely genetic and environmental mechanisms and evidence underlying an association between bone health and LBP [7]. For example, longitudinal data demonstrate the association between cumulative physical activity and bone mass [12], yet the experience of CLBP is often associated with physical disability and reduced vigorous physical activity [13] which are likely to decrease the normal physiologic loads transferred to bone tissue through a de-conditioning syndrome.

Few studies in that review were designed with a primary aim of examining the association between back pain and bone health in middle-aged, healthy adults. Although some evidence for an association between back pain and impaired bone health was identified, comparability between the studies to define a web of evidence was limited due to poor characterisation of LBP amongst the cohorts described, particularly with respect to pain duration, severity and disability. Many inconsistencies in the bone densitometry methods used were also identified as limitations in the literature [7]. For example, bone health is unlikely to be influenced by non-chronic LBP episodes (< 3 months duration) and this may be one reason why studies which examine cohorts with a point prevalence of LBP are unable to establish a relationship between bone density and LBP. Further, the skeletal implications of LBP are likely to be site-specific for a range of reasons including the unique trunk movement patterns adopted by individuals with CLBP, intervertebral disc degeneration and local inflammation. Measurement of bone mineral parameters at non-vertebral sites is therefore less likely to uncover a relationship between bone health and back pain.

Dual energy X-ray absorptiometry (DXA) is the most common clinical tool used to measure areal bone mineral density (aBMD), an accepted surrogate for bone strength. In the context of CLBP, lateral-projection DXA may be a more appropriate method to measure vertebral bone mineral than postero-anterior (PA) projection DXA. The use of lateral-projection DXA reduces the potentially over-riding influence of spinal degenerative conditions, which elevate the proportion of non-trabecular bone tissue, and isolates the metabolically active trabecular bone of interest from the cortical elements of the vertebral body [14]. Further, more precise measures of vertebral depth are possible using lateral-projection techniques, from which apparent volumetric vertebral bone mineral density (ap.vBMD) can be calculated, and these address some of the comparability issues associated with areal measures. Therefore, the aim of this study was to examine the association between vertebral bone mineral measures acquired using lateral-projection DXA and CLBP in a population of middle-aged adults.

## Methods

### Participants

Participants in the study represent a subset of the Joondalup Spinal Health Study (JSHS) cohort. Approval to conduct the JSHS was granted by Curtin University and Edith Cowan University Human Research Ethics Committees and all participants provided written, informed consent.

The JSHS is a community-based cohort study which aims to explore familial aspects in spinal pain presentations by studying families where no member reported a history of LBP in the previous 12 months (control families) and families where at least one parent and one child reported chronic (≥ 3 months duration either continuously, or intermittently such that pain was experienced at least once per week), disabling LBP within the past month (pain families). 'Disabling LBP' was defined as pain impacting on at least 3 of the following areas: lifting, walking, sitting, sleeping, social interaction, travel, need to take medication, need to see a health professional, consistent with an earlier study [15]. A total of 231 participants were recruited for the JSHS for which full datasets were available for 227 (98.3%) participants. A detailed description of the JSHS and the recruitment method has been reported previously [15].

The sample for the current study was derived from adult participants (N = 151) within the JSHS cohort. The sub-group for this study was generated after several exclusion criteria were applied based on historical risk factors for osteoporosis. These risk factors were assessed via questionnaire, completed by each participant independently at home, in order to minimise the potential confounding effects on DXA measures. This resulted in a final sample size of 29 adults with disabling CLBP and 42 adults with no history of LBP in the last 12 months (N = 71). These criteria for exclusion and the number of participants excluded (n) were:

• Aged greater than 60 years to minimise the potential effect of age-related bone loss and spinal degenerative conditions (n = 3 excluded).

• Currently smoking on all or most days or a history of smoking on all or most days in the last 10 years (n = 20 excluded).

• A period of immobilisation of ≥ 6 weeks within the last 12 months to minimise the deleterious effects of immobilisation on bone mass (n = 4 excluded).

• Health conditions known to affect aBMD other than primary osteoporosis and osteopenia (rheumatoid arthritis, osteomalacia, Padget's disease, Cushing's syndrome, ankylosing spondylitis) to exclude the influence of co-morbidities on aBMD (n = 3 excluded: rheumatoid arthritis [n = 2] and osteomalacia [n = 1]).

• Medications known to affect aBMD (oestrogen, progesterone, bisphosphonates and other osteoporosis therapies) which have been taken for ≥ 6 months to exclude pharmacologically-mediated effects on bone (n = 1 excluded).

• Menarche delayed beyond 16 years of age, cessation of normal menstrual periods prior to the age of 45 other than cases of hysterectomy and women more than 5 years post menopause to exclude the potential deleterious effect of reduced circulating oestrogen on aBMD (n = 4 excluded).

• Adults recruited in the JSHS who failed to meet inclusion criteria for CLBP or control groups (n = 24 excluded).

• Body mass index (BMI) < 18 or > 30 to exclude the influence of low body weight and obesity on aBMD measures and accuracy of densitometer performance (n = 21 excluded).

### Questionnaire data

Demographic and clinical characteristics related to LBP were collected via questionnaires, completed independently by each participant. All participants responded to the modified Nordic Musculoskeletal Pain Questionnaire to ascertain the presence and duration of LBP [16]. Psychological wellbeing was measured using the 21-item Depression Anxiety and Stress Scale (DASS-21) [17]. The psychological correlates of CLBP including depression, anxiety and stress have been linked to reduced bone density and reduced bone turnover among pre-, peri-and postmenopausal women [18]. The DASS-21 has three sub-scales (depression, stress and anxiety) each consisting of seven items measured on a 4-point scale (0-did not apply to me at all, to 3-applied to me very much or most of the time). The internal consistency (α = 0.92-0.95) and reliability (r = 0.65-0.78) of the DASS-21 have been established previously [19]. The volume of weekly, non-work-related vigorous physical activity of at least 10 min duration was assessed using a standard question from the International Physical Activity Questionnaire [20]. All participants indicated whether they were currently taking analgesic or non-steroidal anti-inflammatory medications.

Participants who reported currently experiencing CLBP (pain duration of ≥ 3 months) responded to additional pain-specific questions to characterise the nature of their pain. LBP intensity in the past week was quantified with a numeric pain rating scale (0-10 where 0 = no pain and 10 = extreme pain). The personal impact of LBP was measured by asking the participants to indicate the number of LBP episodes in the last year (1-3, 4-10, > 10 episodes), number of work days missed in the last year (0, 1-2, 3-7, 15-30, 181-365 days), and any interference with normal daily activities and recreational activities (yes/no). LBP-related disability was measured with the Oswestry Disability Index (ODI) [21]. The ODI contains 10 questions, each with six ordinal responses scored as 0-5. The total score is expressed as a percentage with a higher score representing higher disability. Internal consistency (Cronbach's alpha (α)) of the ODI in adults is reported to range from α = 0.71-0.87 [21].

### Physical data

Anthropometric and other physical characteristics were measured in a standardised fashion and in a random order in a University laboratory by trained research officers. Height (cm) was measured for each participant without shoes using a standing stadiometer while mass (kg) was measured using electronic scales. The height and mass data were used to calculate BMI (kg/m^2^). Hip and waist girths (cm) were measured using body girth measuring tapes. Measurement of back muscle endurance (BME) was performed using the Biering-Sorenson test [22]. The test requires participants to hold their trunk horizontal in a prone position unsupported for a maximum of 360 s, while the pelvis and lower limbs remain supported on a plinth. Moderate-high reliability (r = 0.66-0.98), in both LBP and non-LBP populations, has been reported [23]. A Hologic Discovery A densitometer (Hologic, Inc, Bedford, MA, USA) was used to measure bone mineral content (BMC) (g) and aBMD (g/cm^2^) in lumbar spine. Apparent volumetric BMD (ap.vBMD) (g/cm^3^) was also measured in the lumbar spine using the vertebral width-adjustment feature of the Hologic analysis software version 12.4:3. The lumbar spine was scanned with a matched PA-lateral scan sequence in high-definition mode using the rotating C-arm feature of the densitometer. All participants lay supine on the scanner bed with hips and knees flexed to 90°, supported by a Hologic positioning device. The upper limbs remained elevated with palms resting behind the back of the head. Analysis of the scans was performed using the automated method with Hologic analysis software version 12.4:3. The L3 vertebra was chosen as the level of interest since previous work suggests that precision of densitometric parameters is maximised at this level [24]. Further, the potentially over-riding influence of the ribs and ilia at L2 and L4, respectively, are overcome by measuring properties at L3 in isolation. DXA variables of interest included L3 BMC (PA and lateral projection), L3 aBMD (PA and lateral projection), total spine BMC and aBMD (PA projection), and L3 width-adjusted ap.vBMD (lateral projection). The T-scores for the PA-projection total spine parameters were also acquired. Quality control of the scanner was monitored by regularly scanning a Hologic phantom. All scanning and analysis was performed by one of three trained research officers. The reliability of performing PA and lateral scanning of the lumbar spine has been established previously [24,25]. The reproducibility of DXA parameters for densitometer used in the laboratory has been established previously with data from a pilot study for PA-projection aBMD in the lumbar spine indicating excellent short-term reproducibility (%CV: 1.1%).

### Data analysis

Questionnaire-derived and physical characteristics were compared between the CLBP groups and genders using t-tests for continuous data and chi square tests for categorical data. Univariate linear regression models were used to examine the association between vertebral DXA variables and predictor variables including the DASS-21 scores, volume of physical activity and back muscle endurance for males and females separately. Subsequently, multiple linear regression models were used to examine the association between study group (CLBP and no LBP) and vertebral DXA-derived variables using the 'enter' method. Separate models were run for each DXA variable of interest and for each gender. The DXA-variables were defined as the dependent variable for each model while 'group' was defined as a dichotomous predictor variable. Each model also included height, mass and age as constant predictor variables so that any association between a given DXA variable and group was adjusted for these factors which are known to influence BMC and aBMD values. Predictor variables identified as significantly-associated with DXA variables in the univariate analyses were also included in the multivariate models. The regression coefficient (B) in each multivariate model represents the mean difference in each DXA variable between groups, adjusted for age, height and mass and other predictor variables. The proportion of variance in each DXA variable accounted for by all the predictor variables in each model was expressed with an R^2 ^value. Data were analysed SPSS Statistics version 17.0 (SPSS, Inc).

## Results

### Demographic and clinical characteristics

The demographic and clinical characteristics and unadjusted DXA-derived parameters of both groups are summarised in Table 1. In both groups, males were significantly heavier, taller and had a greater waist girth than females. Females with CLBP had significantly lower BME than females without CLBP. There were no other within-group or between-group differences in other demographic or clinical variables. As expected, in both groups males had significantly greater unadjusted total spine BMC, and PA-projection L3 BMC than females (*p *< 0.05). In the no-LBP group, males also had significantly greater lateral-projection L3 BMC than females (*p *< 0.05). T-scores across the cohort for the males and females ranged from -2.3 - 1.5 and -2.5 - 3.8, respectively.

**Table 1:** Demographic and clinical characteristics of the no-LBP and CLBP groups, by gender

	No-LBP group			CLBP group			Mean difference (95% CI) between groups	
**Characteristic**	**male**	**female**	**gender mean difference (95% CI)**	**male**	**female**	**gender mean** **difference (95% CI)**	**Male**	**female**

N (%)	17 (40.5)	25 (59.5)		11 (37.9)	18 (62.1)			

Age (years)	35.8 (14.0)	36.2 (12.0)	-0.3 (-8.5,7.8)	36.2 (15.4)	34.7 (14.3)	1.5 (-10.1, 13.0)	-0.4 (-11.9, 11.2)	1.4 (-6.7, 9.6)

Height (cm)	179.3 (7.1)	163.4 (7.7)	15.9 (11.6, 20.6)*	177.6 (4.4)	165.1 (6.0)	12.5 (8.2, 16.8)*	1.7 (-3.2, 6.6)	-1.7 (-6.1, 2.7)

Mass (kg)	79.1 (13.1)	61.8 (10.4)	17.3 (10.0, 24.6)*	81.3 (8.1)	66.3 (9.1)	15.0 (8.1, 21.9)*	-2.2 (-11.3, 6.9)	-4.5 (-10.7, 1.6)

BMI (kg/m^2^)	24.5 (3.1)	23.1 (3.0)	1.4 (-0.5, 3.4)	25.8 (2.7)	24.3 (3.0)	1.5 (-0.8, 3.8)	-1.3 (-3.6, 1.1)	-1.2 (-3.1, 0.6)

Hip girth (cm)	98.4 (7.2)	96.1 (9.3)	2.3 (-3.1, 7.7)	101.3 (6.2)	100.4 (7.1)	0.9 (-4.5, 6.2)	-2.8 (-8.2, 2.6)	-4.3 (-9.5, 1.0)

Waist girth (cm)	84.4 (8.6)	73.2 (10.3)	11.2 (5.2, 17.4)*	86.6 (8.5)	76.2 (9.3)	10.4 (3.3, 17.4)*	-2.3 (-9.0, 4.6)	-3.1 (-9.3, 3.1)

Back muscle endurance (sec) [range 0-360]	188.8 (49.9)	216.4 (92.6)	-27.6 (-77.4, 22.2)	165.3 (51.9)	148.6 (72.9)	16.7 (-35.1, 68.4)	23.5 (-16.8, 63.8)	67.8 (14.7, 120.8)*

DASS-21 depression [range 0-21]	2.1 (3.8)	2.8 (5.6)	-0.7 (-3.9, 2.4)	4.2 (6.7)	2.1 (2.4)	2.2 (-1.4, 5.7)	-2.2 (-6.3, 1.9)	0.7 (-2.1, 3.6)

DASS-21 anxiety [range 0-21]	1.8 (2.3)	2.5 (4.6)	-0.7 (-3.2, 1.7)	2.9 (2.7)	3.1 (3.4)	-0.2 (-2.7, 2.3)	-1.1 (-3.1, 0.8)	-0.6 (-3.2, 1.9)

DASS-21 stress [range 0-21]	8.7 (7.4)	10.1 (7.0)	-1.4 (-5.9, 3.2)	6.5 (6.2)	8.8 (4.9)	-2.2 (-6.5, 2.0)	2.2 (-3.4, 7.7)	1.3 (-2.6, 5.2)

Days/week of vigorous physical activity (days) [range 0-7]	4.1 (1.9)	3.4 (3.4)	0.8 (-0.3, 1.8)	3.6 (2.3)	3.1 (1.4)	0.4 (-1.0, 1.8)	0.6 (-1.1, 2.2)	0.2 (-0.7, 1.2)

ODI (%)				16.2 (8.7)	15.4 (9.1)	0.7 (-6.3, 7.8)		

LBP history (yrs)				13.3 (10.4)	11.6 (9.9)	1.8 (-6.1, 9.7)		

Intensity of LBP in last week; median (IQR)				4 (2)	5 (3)			

Episodes LBP last year; n (%)								
1-3				0 (0)	0 (0)			
4-10				3 (27.3)	8 (44.4)			
> 10				8 (72.7)	10 (55.6)			

Work days missed with last year; n (%)								
0				9 (81.8)	12 (66.7)			
1-2				2 (18.2)	3 (16.7)			
3-7					3 (16.7)			

Interference with normal activities; % reporting 'yes'				5 (45.5)	8 (44.4)			

Interference with recreational activities; % reporting 'yes'				6 (54.5)	8 (44.4)			

Currently taking analgesic medication; % reporting 'yes'	8 (19.0)	19 (45.2)		8 (27.6)	12 (41.4)			

Currently taking NSAID medication; % reporting 'yes'	0 (0)	1 (2.4)		2 (6.9)	2 (6.9)			

PA L3 BMC (g)	20.89 (3.39)	16.72 (3.24)	4.17 (2.05, 6.28)*	20.03 (1.93)	16.46 (3.55)	3.56 (1.17, 5.96)*	0.86 (-1.46, 3.18)	0.26 (-1.87, 2.38)

PA L3 aBMD (g/cm^2^)	1.13 (0.13)	1.08 (0.14)	0.48 (-0.04, 0.14)	1.11 (0.11)	1.10 (0.19)	0.02 (-0.12, 0.15)	0.02 (-0.08, 0.12)	-0.01 (-0.12, 0.09)

PA total spine BMC (g)	79.07 (12.33)	61.30 (13.05)	17.77 (9.66, 25.88)*	75.79 (8.39)	63.50 (13.98)	12.30 (2.70, 21.88)*	3.28 (-5.46, 12.01)	-2.20 (-10.59, 6.19)

PA total spine aBMD (g/cm^2^)	1.15 (0.12)	1.04 (0.14)	0.06 (-0.02, 0.15)	1.08 (0.10)	1.06 (0.18)	0.02 (-0.11, 0.14)	0.03 (-0.06, 0.12)	-0.02 (-0.12, 0.08)

PA total spine T-score	0.08 (1.28)	-0.06 (1.27)	0.14 (-0.75, 1.03)	-0.08 (0.99)	0.32 (1.63)	-0.40 (-1.66, 0.86)	0.15 (-0.90, 1.21)	-0.38 (-1.35, 0.58)

Lateral L3 BMC (g)	9.42 (1.89)	7.80 (1.74)	1.62 (0.45, 2.79)*	8.71 (1.54)	8.36 (1.64)	0.35 (-0.93, 1.62)	0.72 (-0.69, 2.12)	-0.56 (-1.66, 0.54)

Lateral L3 aBMD (g/cm^2^)	0.82 (0.13)	0.77 (0.11)	0.06 (-0.02, 0.14)	0.74 (0.12)	0.78 (0.10)	-0.04 (-0.12, 0.04)	0.08 (-0.02, 0.18)	-0.02 (-0.08, 0.05)

Lateral L3 ap.vBMD (g/cm^3^)	0.21 (0.03)	0.22 (0.03)	-0.01 (-0.03, 0.01)	0.20 (0.03)	0.23 (0.02)	-0.03 (-0.06, -0.01)	0.02 (-0.01, 0.04)	-0.01 (-0.02, 0.01)

*significant difference (*p *< 0.05)

*DASS-21 *21 item depression, anxiety and stress scale

*ODI *oswestry disability index

*LBP *low back pain

*IQR *inter-quartile range

*NSAID *non-steroidal anti-inflammatory drug

*PA *postero-anterior

*BMC *bone mineral content

*aBMD *areal bone mineral density

ap.vBMD: apparent volumetric bone mineral density

Continuous data are expressed as the mean (SD) and categorical data as the frequency (n) and proportion (%). The mean difference and 95% confidence interval (CI) are calculated within groups and between groups for continuous data. DXA-derived parameters are unadjusted in this table

### Univariate associations

In males, the predictor variables (DASS-21 scores, volume of physical activity and back muscle endurance) were not significantly associated with the DXA variables (PA L3 BMC, PA L3 aBMD, PA total spine BMC, PA total spine aBMD, lateral L3 BMC, lateral L3 aBMD, lateral L3 ap.vBMD) in univariate models (r^2 ^= 0-0.09, *p *= 0.13-0.99). Among females, a significant association was identified between vigorous physical activity and lateral-projection L3 variables (lateral L3 aBMD: r^2 ^= 0.11, *p *= 0.04; lateral L3 ap.vBMD: r^2 ^= 0.10, *p *= 0.04), but no other predictor variables (r^2 ^= 0-0.06, *p *= 0.14-0.97).

### Multivariate associations

**Figure 1:**
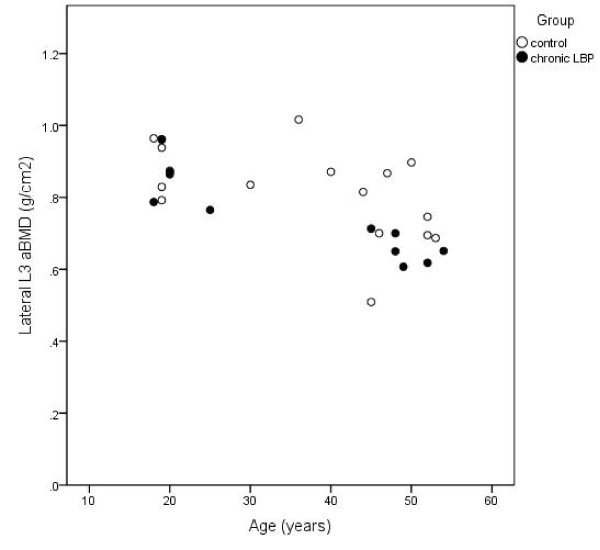
**Unadjusted areal BMD (aBMD) at L3 in males with CLBP and controls according to age**.

**Figure 2:**
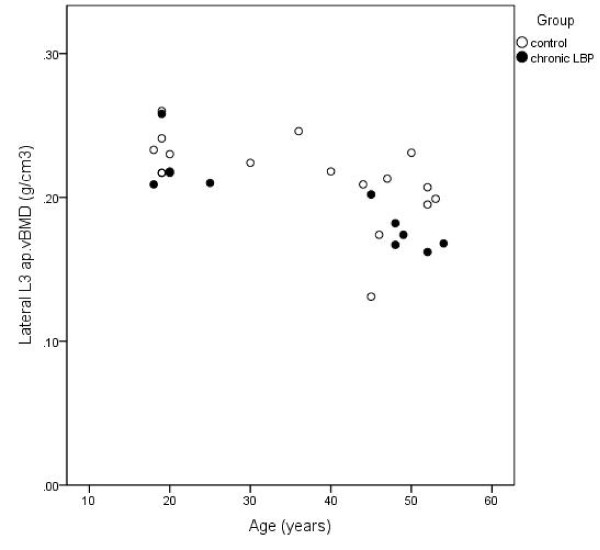
**Unadjusted apparent volumetric BMD (ap.vBMD) at L3 in males with CLBP and controls according to age**.

Multivariate models including the predictor variables CLBP, age, height and mass (and physical activity for some female models) explained between 47 and 55% and 25-35% of the variance in vertebral DXA variables in males and females, respectively (Table 2). A significant association of CLBP with both aBMD and ap.vBMD at L3 measured with lateral-projection DXA was identified in males independent of age, height and mass (Table 2). This association was not observed for DXA-parameters acquired from a PA-projection scan, nor among females. Males with CLBP had significantly lower aBMD and ap.vBMD at L3 compared to males without LBP, with a mean difference (standard error) of 0.09 (0.04) g/cm^2 ^and 0.02 (0.01) g/cm^3^, respectively, when adjusted for covariates. The regression models accounted for 55% and 53% of the variance in lateral-projection aBMD and ap.vBMD at L3, respectively for males. Figures 1 and 2 illustrate the unadjusted L3 aBMD and ap.vBMD, respectively, for males with and without CLBP.

**Table 2:** Results of multiple regression models for the predictor variable 'group' expressed for each DXA dependent variable, adjusted for age, height and mass

	Males				Females			
**DXA variable**	**B co-efficient (group)**	**95% CI**	***p***	**R^2^**	**B co-efficient** **(group)**	**95% CI**	***p***	**R^2^**

PA L3 BMC (g)	-0.78	-3.24, 1.68	0.52	0.11	-0.90	-2.77, 0.96	0.33	0.35^#^

PA L3 aBMD (g/cm^2^)	-0.02	-0.12, 0.09	0.74	0.06	-0.02	-0.12, 0.08	0.73	0.21

PA total spine BMC (g)	-2.89	-11.97, 6.20	0.52	0.14	-0.44	-7.61, 6.73	0.90	0.37^#^

PA total spine aBMD (g/cm^2^)	-0.03	-0.13, 0.07	0.55	0.10	-0.02	-0.11, 0.07	0.71	0.26^#^

Lateral L3 BMC (g)	-0.71	-1.86, 0.45	0.22	0.47^#^	0.24	-0.81, 1.29	0.64	0.25^#^

Lateral L3 aBMD (g/cm^2^)	-0.09	-1.65, -0.01	0.03	0.55^#^	0.01^^^	-0.06, 0.08	0.78^^^	0.21^^^

Lateral L3 ap.vBMD (g/cm^3^)	-0.02	-0.04, -0.001	0.04	0.53^#^	0.01^^^	-0.01, 0.02	0.40^^^	0.16^^^

# multivariate model significant (*p *< 0.05)

^adjusted for physical activity also

*PA *postero-anterior

*BMC *bone mineral content

*aBMD *areal bone mineral density

*ap.vBMD *apparent volumetric bone mineral density

Separate models were run for males (n = 28) and females (n = 43). The unstandardized regression co-efficient (B) represents the mean difference in the DXA variable between the groups, adjusted for the known covariates of age, height and mass with the associated 95% confidence interval (CI) and level of significance (*p*). A negative B value represents a lower DXA parameter in the CLBP group. The R^2 ^represents the proportion of variance in the DXA variables accounted for by all the predictor variables in the model

## Discussion

CLBP in males was associated with reduced vertebral bone mineral measures acquired through lateral-projection DXA, independent of age, height and mass. There are a number of mechanisms which might explain this association, as reported recently [7], yet in this study potential CLNP-related predictor variables of psychological wellbeing, back muscle endurance and physical activity were not associated with bone health, suggesting that other factors may be implicated driving this relationship. These findings raise important questions regarding the mechanisms related to, and clinical impact of, this association and underline the potential clinical utility of lateral-projection DXA methods to assess vertebral bone parameters in some contexts. However, the results presented should be interpreted within the context of the small sample size in the study.

Although previous studies have examined the association between bone mineral measures and LBP, the relationships have been inconsistent, and are likely to be at least partly attributable to suboptimal densitometry methods and sites used. Other studies point to a negative association when bone mineral properties were measured in the lumbar spine [26-29]. For example, Bogdanffy et al [28], in a group of 15 patients (11 male) undergoing L4-S1 spinal fusion for CLBP and lumbar instability observed a significant mean decrease in lateral-projection aBMD at L3 of 10.1% and 11.9% at 3 and 6 months, respectively, after undergoing surgery compared to pre-surgical values. Notably, no significant decrease was observed for aBMD acquired using PA-projection DXA, consistent with our data.

In univariate models, vertebral DXA parameters were not associated with potential predictor variables including the DASS-21 subscales, back muscle endurance and vigorous physical activity (other than in females for some DXA variables). The absence of any associations is likely attributable to the distribution of the predictor variable scores being relatively equal between the CLBP and control groups or insufficient power to identify any differences. This finding suggests that other factors may be important in explaining the association between CLBP and bone health, such as nutrition, other aspects of physical activity (e.g. inactivity), posture, occupation, inflammatory markers and neuroendocrine factors. These data are somewhat contradictory to other reports where individuals with CLBP tend to have poorer psychological wellbeing [30], reduced back muscle endurance [31] and reduced vigorous physical activity [13] relative to those with no pain. The discrepancies may be accounted for by a range of reasons. First, there are differences in the measurement tools between this study and earlier research. Second, the cohort recruited for the JSHS may have predominantly adopted active coping strategies for pain and thus experienced less functional disability. Third, the levels of disability experienced among individuals in this cohort may have been insufficient to significantly influence psychological wellbeing and capacity to engage in vigorous physical activity. Fourth, the sample size was inadequate to detect small, but clinically relevant, associations between the potential predictor variables and the DXA parameters.

The multivariate models demonstrate that in males up to 55% of the variance in vertebral bone mineral parameters acquired using lateral-projection DXA could be accounted for by the combination of age, mass, height and the presence or absence of CLBP. The adjusted mean difference in aBMD and ap.vBMD between the male groups of 0.09 g/cm^2 ^and 0.02 g/cm^3^, respectively represent 0.7 standard deviations below the raw male control group means, or a difference of 11.0% and 9.5%, respectively, comparable to earlier research [28]. This difference appears to be clinically-relevant for this age group in light of the 1.2-2.0 fold increase in the odds of sustaining a vertebral fracture with each 1.0 standard deviation decrease in vertebral aBMD acquired through PA-projection DXA in older men [32,33]. Further, the association between vertebral fracture risk and reduction in bone mineral apparent density (BMAD) acquired using a PA-scan - an apparent volumetric measure similar to ap.vBMD used in our study -is reported to be greater than PA-derived aBMD [33]. Nonetheless, it is acknowledged that reliable vertebral fracture risk estimates have not yet been determined for lateral-projection DXA parameters and therefore the biological significance remains uncertain.

The association between vertebral bone mineral measures and CLBP were only observed for those variables measured using lateral-projection DXA. Evidence continues to emerge to substantiate the potential advantages of lateral-projection methods, such as enhanced diagnostic sensitivity for vertebral fracture [34] and superior predictive capacity for vertebral failure in ex situ models [35]. The lateral approach may better identify reduced bone mineral parameters in the context of CLBP due to the selective inclusion of the metabolically-active trabecular bone. Moreover, age-related degenerative changes in the lumbar spine may obscure any association with CLBP when aBMD is measured using PA-projection methods.

No association between vertebral DXA parameters and CLBP was observed in females. There are a number of potential mechanisms which might explain this gender difference. Although females had lower disability scores, a shorter LBP history, fewer episodes of LBP in the last year, and CLBP causing less interference with recreational activities than males, these gender differences were not statistically-significant and, therefore, are unlikely to account entirely for the absence of an association among females. Hansson et al [29] reported that BMC measured at L3 was negatively associated with the lifetime duration of LBP (years since first onset), and not the severity, disability or duration associated with the current pain episode. Although a greater proportion of males had experienced LBP for ≥ 20 years (27.3% vs. 16.7% in females) in our study, this difference was not statistically significant. The gender difference in the association between CLBP and DXA variables could also be explained by gender-specific neuroendocrine factors, gender differences in the extent of intervertebral disc degeneration, a greater resilience to musculoskeletal pain among females and other osteoporosis risk factors which were not controlled for in this study, such as alcohol and calcium intake and inflammatory markers.

A particular strength of this study is use of both PA-and lateral-projection DXA modalities. Although Bogdanffy et al [28] also used a combination of PA-and lateral-projection DXA, their study was based only on repeated measures in a single group of 15 patients undergoing spinal fusion. To our knowledge, no other studies have used lateral-projection DXA in this context. While lateral DXA may have potential for useful clinical applications, the associations between lateral-projection DXA parameters and fracture risk remain largely unknown, and thus the clinical significance of our findings for vertebral fracture risk are uncertain. Further, inherent limitations exist with DXA, particularly the inability to measure true vertebral volumetric BMD - a parameter which may have demonstrated greater deficits in trabecular BMD between the groups - and indices of bone quality which are equally important as aBMD in mediating bone strength. The association between CLBP and bone quality is currently uncertain, yet this may be an important area for future research, particularly in the context of inflammation-driven back disorders. This study is also limited in the scope of measurement of potentially important correlates of bone health. Although vigorous physical activity was measured, the IPAQ question we used only related to activity undertaken within the previous seven days. More extensive assessments of volumes, frequencies and intensities of vigorous and sedentary activity may be warranted using accelerometry, given the association between CLBP and deficits in vigorous activity [13]. Although several exclusion criteria were applied to this study, we were unable to account for the potentially confounding effects of other important correlates of aBMD including nutrition, inflammatory markers, neuroendocrine markers and other physical factors such as posture, intervertebral disc degeneration and occupation which may influence the association between the presence of CLBP and aBMD. Finally, this study is limited by the small sample size which may increase the risk of a type I error and therefore interpretation of the results presented should be considered within this context. Larger studies should now be undertaken to confirm the findings from this study.

## Conclusions

CLBP in males is associated with some lumbar vertebral BMD measures, raising important questions about the mechanism and potential clinical impact of this association.

## Competing interests

The authors declare that they have no competing interests.

## Authors' contributions

AMB was responsible for conception and design of the study, data collection, data analysis and preparation of the manuscript. LMS, JDW and AFB contributed to the design of the study, procurement of funding to support the study, data collection and preparation of the manuscript. All authors read and approved the final manuscript.

## Pre-publication history

The pre-publication history for this paper can be accessed here:

http://www.biomedcentral.com/1471-2474/13/49/prepub
